# Geographic Analysis of Motor Neuron Disease Mortality and Heavy Metals Released to Rivers in Spain

**DOI:** 10.3390/ijerph15112522

**Published:** 2018-11-11

**Authors:** Germán Sánchez-Díaz, Francisco Escobar, Hannah Badland, Greta Arias-Merino, Manuel Posada de la Paz, Verónica Alonso-Ferreira

**Affiliations:** 1 Institute of Rare Diseases Research (IIER), Instituto de Salud Carlos III, Madrid 28029, Spain; g.sanchez@externos.isciii.es (G.S.-D.); gretaariasmerino@gmail.com (G.A.-M.); mposada@isciii.es (M.P.d.l.P.); 2 Department of Geology, Geography and Environmental Sciences, University of Alcala, Alcalá de Henares 28801, Spain; francisco.escobar@uah.es; 3 Centre for Biomedical Network Research on Rare Diseases (CIBERER), Madrid 28029, Spain; 4 Healthy Liveable Cities Group, Centre for Urban Research, RMIT University, Melbourne VIC 3001, Australia; hannah.badland@rmit.edu.au

**Keywords:** motor neuron disease, population-based mortality, geographical analysis, heavy metals, environmental factors

## Abstract

The etiology of motor neuron disease (MND) is still unknown. The aims of this study were to: (1) analyze MND mortality at a fine-grained level; and (2) explore associations of MND and heavy metals released into Spanish river basins. MND deaths were extracted from the Spanish nationwide mortality registry (2007–2016). Standardized mortality ratios (SMRs) for MND were estimated at a municipal level. Sites that emitted quantities of heavy metals above the regulatory thresholds were obtained from the European Pollutant Release and Transfer Register database (2007–2015). The relative risks for non-exposed and exposed municipalities (considering a downstream 20 km river section) by type of heavy metal were analyzed using a log-linear model. SMRs were significantly higher in central and northern municipalities. SMRs were 1.14 (1.10–1.17) higher in areas exposed to heavy metals than in non-exposed areas: 0.95 (0.92–0.96). Considering the different metals, we found the following increased MND death risks in exposed areas: 20.9% higher risk for lead, 20.0% for zinc, 16.7% for arsenic, 15.7% for chromium, 15.4% for cadmium, 12.7% for copper, and 12.4% for mercury. This study provides associations between MND death risk and heavy metals in exposed municipalities. Further studies investigating heavy metal exposure are needed to progress in MND understanding.

## 1. Introduction

Motor neuron disease (MND) is a neurodegenerative condition in which motor neuron functions diminish progressively in the central nervous system [1]. Currently, there is no cure for MND, and no interventions have been found to stop the progression of symptoms, leading to mortality [2,3]. About 5–10% of MND cases are attributed to familial or genetic origin. Thus, for the remainder of diagnosed patients, environmental factors may have a key role in the development of the disease [4,5,6,7,8].

Numerous studies have examined the effects that metals have in neurodegenerative diseases, focusing especially on the role of heavy metals in health [9,10]. Heavy metal exposure has been associated with amyotrophic lateral sclerosis pathogenesis for more than 150 years, especially since heavy metals were found in tissues and fluids from MND patients [11].

Despite the lack of a general agreement in defining heavy metals, they are typically referred to as those chemical elements having a specific density of more than 5 g/cm^3^ [12]. They are involved in a wide range of processes in various receiving environments such as air, soil, or water. Heavy metal emissions have been continually increasing since the middle of the 19th century at the global level [13].

The association between mercury and MND has been studied, being evident its effects on the human nervous system, leading to brain alterations or to shyness, tremors, irritability, and changes in vision or hearing [14]. Lead pollution, described as toxic for more than 100 years, is a major problem in drinking water pipes in some undeveloped countries, but not in Spain [15]. Chronic exposure to lead has been linked to mental retardation, birth defects, and brain damage, among others [16,17]. Lead is one of the most studied heavy metals in terms of exposure and receiving environment, with results confirming its association with MND [18,19]. Cadmium is a water-soluble heavy metal affecting the kidney and bones. Chromium is highly persistent in water sediments and, in elevated concentrations, it is related to ulcers and can induce DNA damage [20]. A case–control study found an association between cadmium exposure and the occurrence of amyotrophic lateral sclerosis (a type of MND accounting for the 85% of MND cases) in Catalonia, Spain [21]. Copper toxicity can lead to hepatic disorders or neurodegenerative changes [22]. Zinc excesses can cause brain damage or affect the respiratory and gastrointestinal tracts apart from prostate [23], with one of the most common exposing sources being drinking water [24].

At the same time, an increase of MND death rates has been reported in the past years [25,26,27,28]. In Spain, the patterning of MND has been examined, showing geographic variability at the provincial level, but MND mortality has not been further explored at a finer spatial scale throughout the country, and few studies including exposure types have been carried out [18,21,28]. Examining the associations at a more detailed level (e.g., municipality) using geographically referenced environmental data allows a deeper interrogation of the factors that potentially contribute to MND. If a relationship is found between increased MND mortality and heavy metal emissions, it will inform future avenues of inquiry to progress our understanding of MND etiology.

The objectives of this study were to: (1) Analyze the mortality distribution of MND at a municipal level in a 10-year period; (2) Characterize heavy metal emissions released to river basins; and (3) Explore associations between heavy metals exposure in water and MND deaths in Spain over a 10-year period.

## 2. Materials and Methods 

Mortality data were extracted from the annual death registry kept by the National Statistics Institute (NSI) (Instituto Nacional de Estadística), Spain. MND deaths were obtained from 2007 through 2016. These were identified from the International Classification of Diseases, 10th revision, code G12.2, which included all forms of MND. For Standardized Mortality Ratios (SMRs) and confidence intervals (CI) calculations, ages were grouped in five-years intervals up to 84 years old and older. 

Using the European Pollutant Release and Transfer Register (E-PRTR) database, we extracted heavy metal quantities released into river basins by emission point in Spain during 2007–2015, and these were assigned to geographic coordinates provided by this registry [29]. E-PRTR is an information platform, a register with a list of facilities that have emitted polluting substances exceeding certain thresholds. This register contains information about the location, emitted substances, quantities per year, activity, and receiving environment. Facilities are required by law to report the release of pollutants, and this law is implemented by all EU member countries, which have the task to impose penalties on sites with emissions beyond the limits in an effective, proportional, and dissuasive way. Of note, information is only reported by an installation to the E-PRTR when the quantities of pollutants released exceed certain thresholds.

Heavy metals included in this study were: arsenic, cadmium, copper, chromium, mercury, lead, and zinc. These heavy metals were selected because there are several studies linking them to MND symptoms and development [11]. We geocoded the sites that released heavy metal emissions into the rivers during the mentioned period. Using geographic criteria, we identified a 20 km river section downstream from the emission point and selected the municipalities that intersected the sections. These were classified as exposed municipalities. The municipalities that did not intersect these river-sections were defined as non-exposed municipalities.

We then calculated the times that each emission point had exceeded the thresholds by metal as indicated by the E-PRTR. The annual limits per emission point and by metal were: mercury 1 kg; arsenic and cadmium 5 kg; lead 20 kg; chromium and copper 50 kg; Zinc 100 kg [29]. 

We fitted log-linear models on the assumption that the number of MND deaths per stratum followed a Poisson distribution. Observed cases were the dependent variable, and expected cases were included as offset in the models. A term we called “exposure” (distance 20 km or less from the facility) was included as the independent variable. The regression coefficient of this exposure term gave us the logarithm of the ratio between the respective mortality ratios for the exposed and reference zones, which we called incidence report rates (IRR). The advantage of using Poisson regression models is that they provide a magnitude of association (IRR) and a *p*-value of global significance between the exposure variable and mortality.

Statistical analyses were performed using the Stata computer software program, while the ArcGIS software program (Esri, Redlands, CA, USA) was used for geographic analysis and cartographic representations.

### Research Ethics

The study was conducted in accordance with the Declaration of Helsinki, and the protocol was approved by the Ethics Committee of the Instituto de Salud Carlos III (CEI 50/2013).

## 3. Results

**Figure 1:**
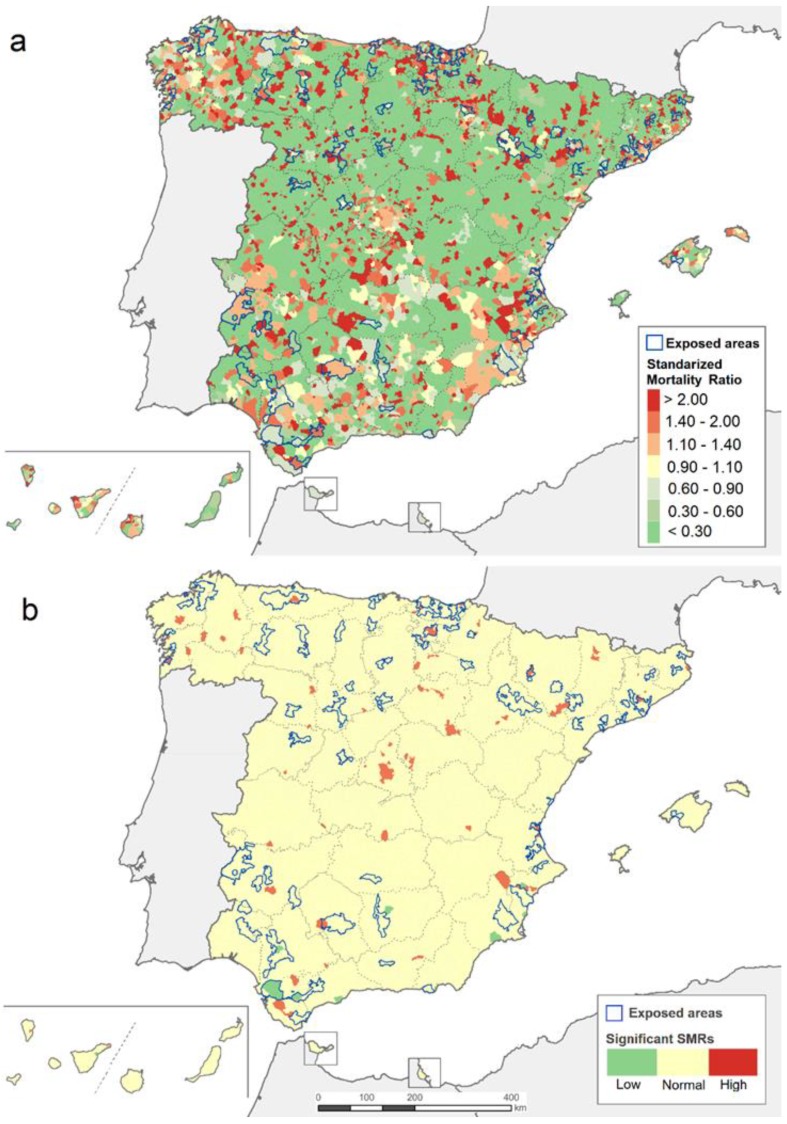
(**a**) Distribution of standardized mortality ratios (SMRs) due to motor neuron disease (MND) from 2007 to 2016. (**b**) Distribution of lower- and higher-than-expected SMRs.

There were 9434 deaths due to MND in the period 2007–2016 in the Spanish territory. As shown in Figure 1a, results obtained for municipal SMRs seemed to be random across the period 2007–2016, when it was possible to observe both high and low SMRs throughout the national territory. Figure 1b depicts only significant SMRs, highlighting 90 municipalities. The majority of lower-than-expected SMRs values (15 municipalities) were located in the southern half of the Spanish territory, whereas the majority of the higher-than-expected SMRs results (75 municipalities) were located in the northern half of the country.

**Figure 2:**
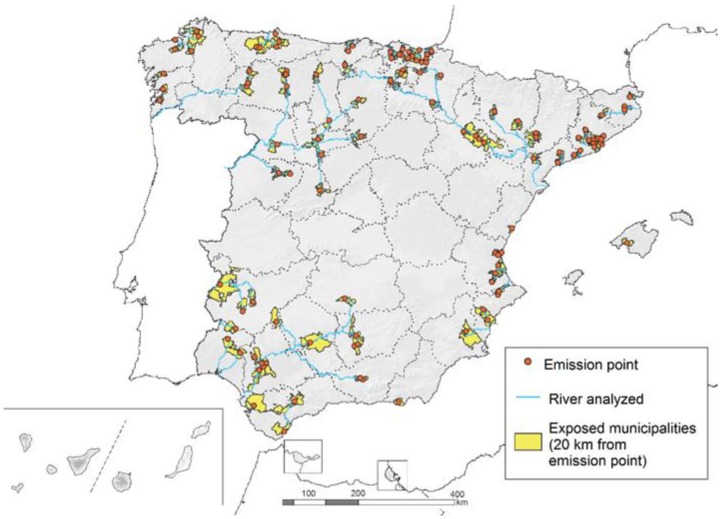
Location of sites (emission point) releasing heavy metals to river basins in the period 2007–2015 in Spain.

We identified 129 sites that reported heavy metal quantities emitted to river basins that exceeded the thresholds during the period 2007–2015 (Figure 2), noting that a site may release more than one type of heavy metal. Overall, 92 sites were located in the northern part of Spain, mainly in Basque Country and Catalonia. Zinc was the most commonly reported exceeding heavy metal emission, whilst cadmium was the least (99 and 23 sites, respectively). The main activities identified at the sites were: wastewater treatment sludge, thermal power stations, steel industry, paper industry, and chemical industry. Overall, 458 municipalities were affected (5.64% of the total Spanish municipalities) as they intersected 20 km of river downstream from the sites. According to the location of the emission point for each heavy metal, the number of exposed municipalities varied: zinc *n* = 382, copper *n *= 235, arsenic *n* = 198, mercury *n* = 169, lead *n* = 163, chromium *n *= 156, and cadmium *n*=93.

The results of the Poisson Regressive Model used to compare mortality due to MND in exposed versus non-exposed municipalities is shown in Table 1. Significant SMR differences between the exposed and non-exposed municipalities existed. However, SMR attributed to MND was significantly higher in exposed municipalities for all metals and for individual metals. The IRR were higher in exposed than in non-exposed municipalities (*p* < 0.001), with an 18.4% increased risk of MND for people living in municipalities exposed to heavy metals. MND risk varied by metal exposure—lead posed a 20.9% risk (highest), whereas copper and mercury increased the risk by 12.4% (lowest).

**Figure 3:**
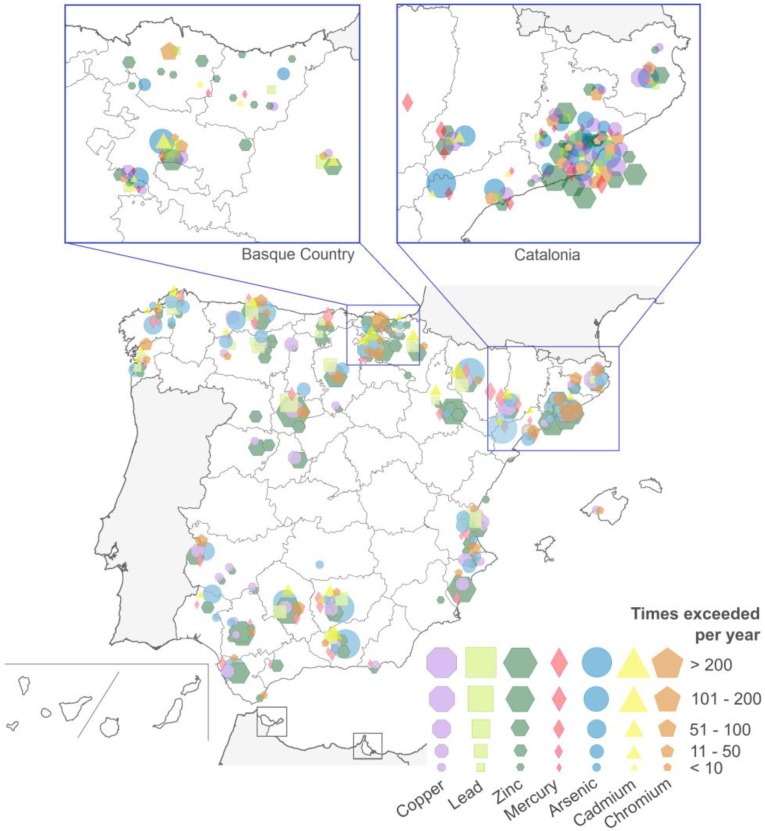
Number of times that heavy metal emissions exceeded the annual threshold by heavy metal and per site in 2007–2015. Only the emission points that in at least one year exceeded the maximum regulatory emission level are displayed.

Comparing the quantities of heavy metal emissions with the EU regulatory thresholds, we observed that zinc was the most common substance emitted exceeding its limit (100 kg per year per site), as shown in Figure 3. Across many emission points, the annual threshold of zinc was exceeded by more than 100, 200, or even 500 times. Cadmium and lead were the heavy metals least likely to exceed the regulatory thresholds, with exceeding values measured up to maximum 100 times per year per site as during the period 2007–2015.

## 4. Discussion

This study provides a detailed map of MND mortality in Spain by exploring possible associations between the MND and heavy metal exposure. The discovery of patterning of significant high and low mortality rates through the Spanish territory supports a non-randomly spatial distribution of MND. This spatial pattern is in line with and extends from other MND studies conducted at a provincial level [18,28]. The increased MND risk identified in certain municipalities close to heavy metal emissions suggests that environmental factors may contribute to MND etiology.

Much research supports the notion of heavy metals being harmful to health, with effects varying according to dose, duration, exposure conditions, bioavailability, and chemical species [11,30]. Despite different studies attempting to rank the health effects of specific heavy metals, a consensus has not been obtained. For example, the U.S. Department of Health and Human Services, Agency for Toxic Substances and Disease Registry (ATSDR), recognized arsenic, lead and mercury as being the heavy metals most harmful to human health, on the basis of a combination of frequency and potential exposure [31]. In relation to specific heavy metals, we found that municipalities exposed to lead had the highest increased MND risk (20.9%), while those exposed to mercury showed the lowest increased risk (12.4%) in reference to non-exposed areas. 

Although pollutant emission data were available from 2001, we only used data from 2007; in the period 2001–2007, it was not necessary for emission points to report the exceeding quantities. The register was further improved in 2007, with more substances included and a better evaluation of activities occurring at the sites [32]. The strengths of this study are the potential extension of this method to include information on other substances and the receiving environment (e.g., air, soil) and an exhaustive analysis of the activity of sites and the measurements taken after the thresholds were exceeded. The database from which we extracted the heavy metal emissions data are also available for other 34 EU countries. The possibility of collecting national MND deaths registries, standardized and comparable across countries, and the study of heavy metal emissions in a continental context would provide a large-scale study with large-scale exposure heterogeneity. Identifying associations in a wider geographic context would be very useful for the European health policy planning and for further identifying the etiology of and risk factors for MND. Our paper only focuses on the likely effects of isolated metals on MND mortality and not on the synergistic or antagonistic effects of mixing various types of metals in water. For instance, there are several articles relating the severe effects in mice of metals like cadmium combined with fluoride dissolved in water [33]. These possible synergistic effects should be studied more deeply in the future.

There are limitations in this study. The mortality statistics from NSI did not provide individual information about personal habits, disease-related genetic history, or occupation. We did not have a direct measurement of individual exposure to heavy metals. Another limitation is that this registry does not include information about the possible movement of patients from small to larger municipalities, once diagnosed, for better treatment. This would imply that MND deaths could be inflated in big cities, while underestimated in small municipalities. These changes in the municipality of residence could result in less accuracy when exploring likely associations. However, the population-based and standardized mortality data used are a noticeable advantage because the registry has national coverage.

Exposure to metals is, in most cases, a long-term process; it would, therefore, be valuable to have reliable and standardized data about pollutant emissions available for longer time periods (i.e., decades). This information could be used to better assess and understand the association between neurodegenerative diseases and pollutant exposures. It should be noted that sites are not required to report quantity emissions when they do not exceed the set threshold; therefore, when we found a gap in a year information in one emission point in the studied period, it could mean two things: a released quantity lower than the limit or no emission produced. The absence of data seems to indicate that, in the event of heavy metal release, this would not involve a health hazard. Finally, it is important to note that this is emergent research that needs to be analyzed with greater precision in future studies, especially as regards the interaction of heavy metals with the rivers where they are released in terms of dissolution of substances in water and hydraulic flow of each river, and the study of multiple cross-sectional distances from the emission points.

While this study does not definitively link heavy metal exposure to MND, it identifies a valid approach and further lines of inquiry. Within a national context, this is a novel study which indicates characteristics of MND mortality and heavy metals that suggest further investigations at a local level, considering river characteristics and the behaviors of the different heavy metal in rivers.

## 5. Conclusions

In conclusion, we described the detailed mortality rates due to MND during 10 years in Spain, finding patterns that indicate higher rates than expected of MND in northern municipalities. Also, we explored heavy metal emissions in river basins, identifying emission points exceeding the recommended thresholds predominantly located in northern areas. Increased MND mortality risk in the exposed areas was shown when analyzing heavy metals individually and together. This investigation underscores the value of combining geographic and epidemiologic techniques for the understanding of the patterning of relationships between rare diseases and environmental exposures.

## Figures and Tables

**Table 1:** Comparison of motor neuron disease mortality in exposed and non-exposed municipalities based on individual heavy metals: number of deaths (%), SMR, and incidence report rate (IRR). MND: motor neuron disease SMR: Standardized mortality ratio.

	Non-Exposed Municipalities		Exposed Municipalities		Comparison between Non-Exposed vs. Exposed Municipalities			
	Deaths from MND (%)	SMR	Deaths from MND (%)	SMR	IRR	95% CI	*p*-Value	Increased Risk
All heavy metals	6158 (65.27)	0.95 (0.92–0.96)	3276 (34.73)	1.14 (1.10–1.17)	1.184	1.135–1.236	<0.001	18.4%
Arsenic	7348 (77.89)	0.97 (0.95–0.99)	2086 (22.11)	1.13 (1.08–1.18)	1.167	1.112–1225	<0.001	16.7%
Cadmium	8235 (87.29)	0.98 (0.96–1.00)	1199 (12.71)	1.14 (1.07–1.20)	1.154	1.086–1.227	<0.001	15.4%
Chromium	7366 (78.08)	0.97 (0.95–0.99)	2068 (21.92)	1.12 (1.08–1.17)	1.157	1.102–1.215	<0.001	15.7%
Copper	7231 (76.65)	0.97 (0.95–1.00)	2203 (23.35)	1.10 (1.05–1.14)	1.127	1.075–1.182	<0.001	12.7%
Lead	7533 (79.34)	0.97 (0.94–0.99)	1901 (20.66)	1.17 (1.12–1.22)	1.209	1.150–1.271	<0.001	20.9%
Mercury	7397 (78.41)	0.98 (0.95–1.00)	2037 (21.59)	1.10 (1.05–1.15)	1.124	1.071–1.181	<0.001	12.4%
Zinc	6309 (66.88)	0.94(0.92–0.97)	3125 (33.12)	1.13 (1.09–1.17)	1.200	1.150–1.253	<0.001	20.0%

## References

[1] Kiernan M.C. (2018). Motor neuron disease in 2017: Progress towards therapy in motor neuron disease. Nat. Rev. Neurol..

[2] Mehta P., Kaye W., Raymond J., Wu R., Larson T., Punjani R., Heller D., Cohen J., Peters T., Muravov O., Horton K. (2018). Prevalence of amyotrophic lateral sclerosis—United States, 2014. MMWR Suppl..

[3] Miller R.G., Appel S.H. (2017). Introduction to supplement: The current status of treatment for ALS. Amyotroph. Lateral Scler. Frontotemporal Degener..

[4] Rosen D.R., Siddique T., Patterson D., Figlewicz D.A., Sapp P., Hentati A., Donaldson D., Goto J., O’Regan J.P., Deng H.X. (1993). Mutations in Cu/Zn superoxide dismutase gene are associated with familial amyotrophic lateral sclerosis. Nature.

[5] Strong M., Rosenfeld J. (2003). Amyotrophic lateral sclerosis: A review of current concepts. Amyotroph. Lateral Scler. Other Motor Neuron Disord..

[6] Kiernan M.C., Vucic S., Cheah B.C., Turner M.R., Eisen A., Hardiman O., Burrell J.R., Zoing M.C. (2011). Amyotrophic lateral sclerosis. Lancet.

[7] Ingre C., Roos P.M., Piehl F., Kamel F., Fang F. (2015). Risk factors for amyotrophic lateral sclerosis. Clin. Epidemiol..

[8] Huisman M.H., de Jong S.W., van Doormaal P.T., Weinreich S.S., Schelhass H.J., van der Kooi A.J., de Visser M., Veldink J.H., van den Berg L.H. (2011). Population based epidemiology of amyotrophic lateral sclerosis using capture-recapture methodology. J. Neurol. Neurosurg. Psychiatry.

[9] Barnham K.J., Bush A.I. (2014). Biological metals and metal-targeting compounds in neurodegenerative diseases. Chem. Soc. Rev..

[10] Jaishankar M., Tsseten T., Anbalagan N., Mathew B.B., Beeregowda K.N. (2014). Toxicity, mechanism and health effects of some heavy metals. Interdiscip. Toxicol..

[11] Roos P.M. Studies on Metals in Motor Neuron Disease. https://openarchive.ki.se/xmlui/handle/10616/41419.

[12] Järup L. (2003). Hazard of heavy metal contamination. Br. Med. Bull..

[13] Nragu J.O. (1996). History of global metal pollution. Science.

[14] Praline J., Guennoc A., Limousin N., Hallak H., de Toffoi B., Corcia P. (2007). Case report; ALS and mercury intoxication: A relationship?. Clin. Neurol. Neurosurg..

[15] Etabe I.Z., Contín K.C., Olalde C.O., Alonso J.V. (2010). Release of lead and other metals from piping into drinking water in the Basque Country (Spain). Gac. Sanit..

[16] Brochin R., Leone S., Phillips D., Shepard N., Zisa D., Angerio A. (2008). The cellular effect of lead poisoning and its clinical picture. GUJHS.

[17] Campbell A.M., Williams E.R., Barltrop D. (1970). Motor neuron disease and exposure to lead. J. Neurol. Neurosurg. Psychiatry.

[18] Santurtún A., Villar A., Delgado-Alvarado M., Riancho J. (2016). Trends in motor neuron disease: Association with air lead levels in Spain. Neurol. Sci..

[19] Kamel F., Umbach D.M., Hu H., Munsat T.L., Shefner J.M., Taylor J.A., Sandler D.P. (2005). Lead exposure as a risk factor for amyotrophic lateral sclerosis. Neurodegener. Dis..

[20] Bernard A. (2008). Cadmium and its adverse effects on human health. Indian J. Med. Res..

[21] Povedano M., Saez M., Martínez-Matos J., Barceló M.A. (2018). Spatial assessment of the association between long-term exposure to environmental factors and the occurrence of Amyotrophic Lateral Sclerosis in Catalonia, Spain: A population-based nested Case-Control study. Neuroepidemiology.

[22] Gaetke L.M., Chow-Johnson H., Chow C.K. (2014). Copper: Toxicological relevance and mechanisms. Arch. Toxicol..

[23] Plum L.M., Rink L., Haase H. (2010). The essential toxin: Impact of Zinc on human health. Int. J. Environ. Res. Public Health.

[24] (2006). Agency for Toxic Substances & Disease Registry (ATSDR): ToxFAQs for ZINC. https://www.atsdr.cdc.gov/toxprofiles/TP.asp?id=302&tid=54.

[25] Sejvar J.J., Holman R.C., Bresee J.S., Kochanek K.D., Schonberger L.B. (2005). Amyotrophic lateral sclerosis mortality in the United States, 1979–2001. Neuroepidemiology.

[26] Nakken O., Lindstrøm J.C., Tysnes O.B., Holmøy T. (2016). Mortality trends of amyotrophic lateral sclerosis in Norway 1951–2014: An age-period-cohort study. J. Neurol..

[27] Chiò A., Logroscino G., Traynor B.J., Collins J., Simeone J.C., Goldstein L.A., White L.A. (2013). Global epidemiology of amyotrophic lateral sclerosis: A systematic review of the published literature. Neuroepidemiology.

[28] Alonso V., Villaverde-Hueso A., Hens M., Morales-Piga A., Abaitua I., de la Paz M.P. (2011). Increase in motor neuron disease mortality in Spain: Temporal and geographical analysis (1990–2015). Amyotroph. Lateral Scler..

[29] European Parliament (2006). Regulation (EC) No 166/2006 of the European Parliament and of the Council of 18 January 2006 Concerning the Establishment of a European Pollutant Release and Transfer Register and Amending Council Directives 91/689/ECC and 96/61/EC.

[30] Tchounwou P.B., Yedjou C.G., Patlolla A.K., Sutton D.J. (2012). Heavy metal toxicity and the environment. Molecular, Clinical and Environmental Toxicology.

[31] Agency for Toxic Substances and Disease Registry Substance Priority List-ATSDR. https://www.atsdr.cdc.gov/spl/.

[32] García-Pérez J., Boldo E., Ramis R., Pollán M., Pérez-Gómez B., Aragonés N., López-Abente G. (2007). Description of industrial pollution in Spain. BMC Public Health.

[33] Wasana H.M., Perera G.D., Gunawardena P.S., Fernando P.S., Bandara J. (2017). WHO water quality standards vs. Synergic effect(s) of fluoride, heavy metals and hardness in drinking water on kidney tissues. Sci. Rep..

